# Enzymes Catalyzing Crotonyl-CoA Conversion to Acetoacetyl-CoA During the Autotrophic CO_2_ Fixation in *Metallosphaera sedula*

**DOI:** 10.3389/fmicb.2020.00354

**Published:** 2020-03-11

**Authors:** Li Liu, Harald Huber, Ivan A. Berg

**Affiliations:** ^1^Institute for Molecular Microbiology and Biotechnology, University of Münster, Münster, Germany; ^2^Institute for Microbiology and Archaeal Center, University of Regensburg, Regensburg, Germany

**Keywords:** 3-hydroxypropionate/4-hydroxybutyrate cycle, Sulfolobales, *Metallosphaera sedula*, crotonyl-CoA hydratase, 3-hydroxybutyryl-CoA dehydrogenase

## Abstract

Autotrophic Crenarchaeota use two different cycles for carbon dioxide fixation. Members of the Sulfolobales use the 3-hydroxypropionate/4-hydroxybutyrate (HP/HB) cycle, whereas Desulfurococcales and Thermoproteales use the dicarboxylate/4-hydroxybutyrate cycle. While these two cycles differ in the carboxylation reactions resulting in the conversion of acetyl-CoA + 2 CO_2_ to succinyl-CoA, they have a common regeneration part in which succinyl-CoA is reconverted to two acetyl-CoA molecules. This common part includes crotonyl-CoA conversion to acetoacetyl-CoA, which has unequivocally been shown in *Ignicoccus hospitalis* (Desulfurococcales) and *Pyrobaculum neutrophilus* (Thermoproteales) to be catalyzed by a bifunctional crotonase/3-hydroxybutyryl-CoA dehydrogenase. It is a fusion protein consisting of an enoyl-CoA hydratase and a dehydrogenase domain. As the homologous bifunctional protein is present in Sulfolobales as well, its common functioning in the conversion of crotonyl-CoA to acetoacetyl-CoA was proposed. Here we show that a model autotrophic member of Sulfolobales, *Metallosphaera sedula*, possesses in addition to the bifunctional protein (Msed_0399) several separate genes coding for crotonyl-CoA hydratase and (*S*)-3-hydroxybutyryl-CoA dehydrogenase. Their genes were previously shown to be transcribed under autotrophic and mixotrophic conditions. The dehydrogenase Msed_1423 (and not the bifunctional protein Msed_0399) appears to be the main enzyme catalyzing the (*S*)-3-hydroxybutyryl-CoA dehydrogenase reaction. Homologs of this dehydrogenase are the only (*S*)-3-hydroxybutyryl-CoA dehydrogenases present in all autotrophic Sulfolobales, strengthening this conclusion. Two uncharacterized crotonase homologs present in *M. sedula* genome (Msed_0336 and Msed_0384) were heterologously produced and characterized. Both proteins were highly efficient crotonyl-CoA hydratases and may contribute (or be responsible) for the corresponding reaction in the HP/HB cycle *in vivo*.

## Introduction

Autotrophic CO_2_ fixation is the most important biosynthetic process in nature, being responsible for the primary production of organic matter. Autotrophy is arguably the oldest type of metabolism (Wächtershäuser, 1988; Russell and Martin, 2004; Fuchs, 2011), and the first metabolic pathway was probably the (autotrophic) reductive acetyl-CoA pathway (Russell and Martin, 2004; Fuchs, 2011; Weiss et al., 2016). During evolution, several fundamentally different CO_2_ fixation pathways evolved. They vary not only in their reaction sequences, but also in their types of carboxylases, cofactors, and electron donors used to fix inorganic carbon into biomass (Berg et al., 2010a; Berg, 2011; Fuchs, 2011; Hügler and Sievert, 2011). Conversion of CO_2_, a greenhouse gas, into value-added products attracts great attention to autotrophic metabolism, rendering it one of the popular topics for fundamental and applied research.

**FIGURE 1:**
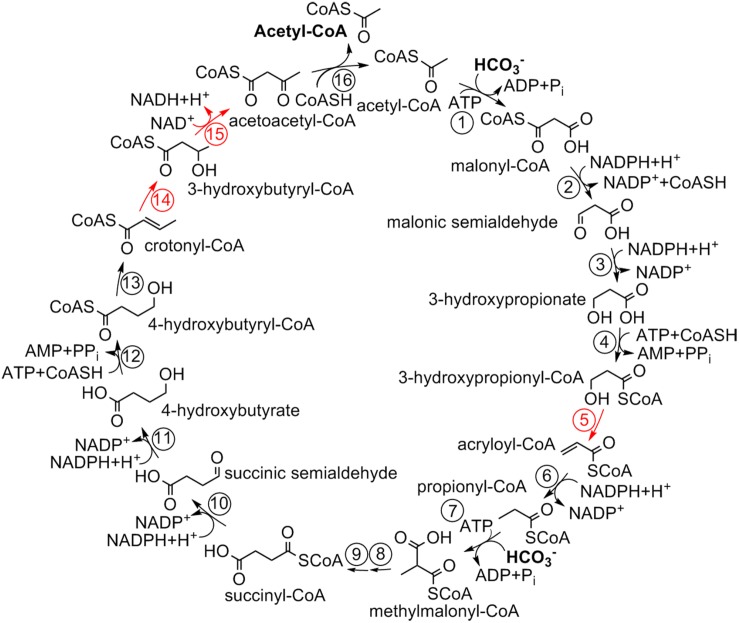
3-Hydroxypropionate/4-hydroxybutyrate cycle in Sulfolobales (Berg et al., 2007). For the enzymes, see Table 1.

The 3-hydroxypropionate/4-hydroxybutyrate (HP/HB) cycle was discovered in thermophilic aerobic Crenarchaeota of the order Sulfolobales (Figure 1; Ishii et al., 1996; Menendez et al., 1999; Berg et al., 2007). Later, a formally similar, but phylogenetically unrelated pathway was shown in aerobic ammonia-oxidizing Thaumarchaeota (Könneke et al., 2014; Otte et al., 2015). While the autotrophic cycle in Thaumarchaeota is less studied, with some characteristic enzymes of the cycle still being unidentified, all enzymes of the crenarchaeal cycle were identified and biochemically characterized (Table 1).

**TABLE 1:** Overview of the enzymes involved in the 3-hydroxypropionate/4-hydroxybutyrate cycle of autotrophic CO_2_ assimilation in *M. sedula*.

Reaction (Nr. in Figure 1)	Genes, according to Berg et al. (2007)	Genes, according toLoder et al. (2016)	Min. act. in cell extract(Berg et al., 2007)^a^	*V*_max_ or specificactivity, μ mol min^–1^ mg^–1^ protein	Regulation: auto/mixo. versus heterotr^b^	Min.% of cellular protein^c^	References
Acetyl-CoA carboxylase (1)	Msed_0147/Msed_0148/Msed_1375	Msed_0147/Msed_0148/Msed_1375	0.11	–/3.2^d,e^	Up	3.4	Hügler et al., 2003

Malonyl-CoA reductase (2)	Msed_0709	Msed_0709	0.42	–/44^f,g^ –/40^f,g^	Up	0.5	Kockelkorn and Fuchs, 2009; Loder et al., 2016

Malonic semialdehyde reductase (3)	Unknown	Msed_1993	3.0	–/200^f,g^	Up	0.5	Kockelkorn and Fuchs, 2009

3-Hydroxypropionyl-CoA synthetase (4)	Msed_1456	Msed_1456	0.24	18^g^/–	Up	0.7	Alber et al., 2008

3-Hydroxypropionyl-CoA dehydratase (5)	Msed_2001	Msed_2001	0.24	–/151^f,g^ –/272^f,g^	No	0.04	Teufel et al., 2009; Loder et al., 2016

Acryloyl-CoA reductase (6)	Msed_1426	Msed_1426	0.24	–/7.6^f,g^ –/18.7^f,g,h^	Up	0.9	Teufel et al., 2009; Loder et al., 2016

Propionyl-CoA carboxylase (7)	Msed_0147/Msed_0148/Msed_1375	Msed_0147/Msed_0148/Msed_1375	0.12	–/3.3^d,e^	Up	3.6	Hügler et al., 2003

Methylmalonyl-CoA epimerase (8)	Msed_0639	Msed_0639	0.06^i^	–/218^f,g^	(Up)	0.1	Han et al., 2012

Methylmylonyl-CoA isomerase (9)	Msed_0638/Msed_2055	Msed_0638/Msed_2055	0.06^i^	–/2.2^f,g^	Up	1.4	Han et al., 2012

Succinyl-CoA reductase (10)	Msed_0709	Msed_0709	1.0	–/40^f,g^	Up	1.3	Kockelkorn and Fuchs, 2009

Succinic semialdehyde reductase (11)	Msed_1424	Msed_1424	5.0	–/700^f,g^ –/683^f,g^	Up	0.4	Kockelkorn and Fuchs, 2009; Loder et al., 2016

4-Hydroxybutyryl-CoA synthetase (12)	Msed_1422	Msed_0406	0.3	ND^j^ –/1.7^f,h^	Up No	12.5	Berg et al., 2007; Hawkins et al., 2013a, 2014

4-Hydroxybutyryl-CoA dehydratase (13)	Msed_1220 Msed_1321	Msed_1321	0.39	ND –/2.2^f,h^	No Up	12.5	Hawkins et al., 2014

Crotonyl-CoA hydratase (14)	Msed_0399 Msed_0384 Msed_0385 Msed_0336 Msed_0566	Msed_0399	15.0	13.8^f^ ^,g^/38^g^ –/20^f,h^ ND ND ND ND	No Down Down No No	19.7	Ramos-Vera et al., 2011; Hawkins et al., 2014

(*S*)-3-Hydroxybutyryl-CoA dehydrogenase (15)	Msed_1423 Msed_0399 Msed_1993^I^ Msed_0389	Msed_0399	1.6	ND –/16^f,h^ –^l^ ND	(Up) No – Up	(2.1)^k^	Kockelkorn and Fuchs, 2009; Hawkins et al., 2014

Acetoacetyl-CoA ß-ketothiolase (16)	Msed_0656 Msed_1647 Msed_1290 Msed_0396 Msed_0386 Msed_0271 Msed_0270	Msed_0656	1.06	–/141^f,h^ ND ND ND ND ND ND	Up ? Down Down Up ? ?	0.5	Hawkins et al., 2014

Summe	–	–	–	–	–	58.04	–

*^a^Minimal activity in cell extracts of M. sedula was tentatively extrapolated to 75°C. ^b^Up-regulation in mixotrophically and autotrophically grown cells, based on transcriptomic analysis (Auernik and Kelly, 2010; Hawkins et al., 2013a; Ai et al., 2019); up, clear up-regulation; (up), slightly up-regulatred; down, down-regulated; no, no regulation; –, not applicable; ?, no obvious trend. ^c^The relative abundance of individual protein in the cell (in %) was calculated as (enzymatic activity in cell extract, extrapolated to 75°C/specific activity or V_max_ of the corresponding enzyme, extrapolated to 75°C) × 100%. ^d^Native purification. ^e^Activity at 75°C. ^f^Heterologously produced protein. ^g^Activity at 65°C. ^h^Activity at 70°C. ^i^Minimal in vivo specific activity of enzymes in the cycle, calculated based on the growth rate (Berg et al., 2007). ^j^Enzyme identification by partial purification. ^k^Bifunctional crotonyl-CoA hydratase/3-hydroxybutyryl-CoA dehydrogenase was taken into account in the calculation for the crotonyl-CoA hydratase reaction. ^l^Malonic semialdehyde reductase.*

In former studies, the model crenarchaeon *Metallosphaera sedula* was used; it is capable of growing autotrophically with hydrogen, sulfidic ores or S^0^ under aerobic conditions. Furthermore, heterotrophic growth with, e.g., peptone or yeast extract and mixotrophic growth in the presence of organic compounds and inorganic electron donors is possible. The transcriptomic studies of *M. sedula* revealed that most of the enzymes of the cycle were up-regulated under mixotrophic and autotrophic growth conditions (Auernik and Kelly, 2010; Hawkins et al., 2013a; Ai et al., 2019). Nevertheless, there were several notable exceptions, as 3-hydroxypropionyl-CoA dehydratase, 4-hydroxybutyryl-CoA synthetase and bifunctional crotonyl-CoA hydratase/3-hydroxybutyryl-CoA dehydrogenase were not regulated, or even down-regulated in the presence of inorganic electron donors (Table 1; Auernik and Kelly, 2010; Hawkins et al., 2013a; Ai et al., 2019). Two of these enzymes, 4-hydroxybutyryl-CoA synthetase and bifunctional crotonyl-CoA hydratase/3-hydroxybutyryl-CoA dehydrogenase, stand out in comparative analysis of specific activities of the purified enzymes with the corresponding activities in cell extracts of *M. sedula*. This analysis allows to estimate the amount of the individual enzyme proteins required to account for autotrophic growth, based on the specific activities of the purified enzymes and the measured specific enzyme activities in cell extract. This estimate led to the surprising high number of 58% of total cellular protein required to operate the HP/HB cycle (Table 1). 4-Hydroxybutyryl-CoA synthetase and crotonyl-CoA hydratase/3-hydroxybutyryl-CoA dehydrogenase alone were responsible for more than half of this amount (12.5 and 19.7%, respectively). Whereas 4-hydroxybutyryl-CoA dehydratase appears to constitute a large fraction of cellular protein as well, it slowly catalyzes a mechanistically difficult radical reaction (Martins et al., 2004; Buckel, 2019) and is indeed present in high amounts in other organisms (Scherf and Buckel, 1993). Furthermore, the posttranscriptional inactivation of AMP-producing organic acid CoA-ligases by acetylation has been frequently reported and may result in down-estimation of the activity of recombinant 4-hydroxybutyryl-CoA synthetase (Crosby et al., 2010; Ramos-Vera et al., 2011). However, crotonyl-CoA hydratase catalyzes a relatively simple reaction, and its postulated high abundance in the cell is surprising.

Here we studied enzymes catalyzing the conversion of crotonyl-CoA to acetoacetyl-CoA in *M. sedula*. We found that this conversion might be catalyzed by a number of isoenzymes with different kinetic properties, and not by a single bifunctional enzyme, as was previously thought.

## Materials and Methods

### Materials

Chemicals and biochemicals were obtained from Fluka (Neu-Ulm, Germany), Sigma-Aldrich (Deisenhofen, Germany), Merck (Darmstadt, Germany), VWR (Darmstadt, Germany), Roth (Karlsruhe, Germany), Applichem (Darmstadt, Germany), IBA (Göttingen, Germany) or Cell Signaling Technology (Frankfurt, Germany). Materials for cloning and expression were purchased from MBI Fermentas (St. Leon-Rot, Germany), New England Biolabs (Frankfurt, Germany), and Novagen (Schwalbach, Germany). Materials and equipment for protein purification were obtained from GE Healthcare (Freiburg, Germany), Macherey-Nagel (Düren, Germany), Pall Corporation (Dreieich, Germany) or Millipore (Eschborn, Germany). Primers were synthesized by Sigma-Aldrich (Steinheim, Germany).

### CoA-Esters

Crotonyl-CoA and methacrylyl-CoA were synthesized from the corresponding anhydrides and CoA by the method of Simon and Shemin (1953). (*S*)-3-hydroxybutyryl-CoA, (*R*)-3-hydroxybutyryl-CoA, and (*E*)-2-octenoyl-CoA were synthesized chemically from the corresponding free acids by the mixed anhydride method described by Stadtman (1957). 3-Hydroxypropionyl-CoA was synthesized enzymatically with recombinant propionate CoA-transferase from *Clostridium propionicum* as described by Selmer et al. (2002). Acryloyl-CoA was synthesized enzymatically with recombinant acyl-CoA oxidase 4 from *Arabidopsis thaliana* as described in Schwander et al. (2016). Crotonyl-CoA, 3-hydroxybutyryl-CoA, and (*E*)-2-octenoyl-CoA were purified using HPLC (Zarzycki et al., 2009). The dry powders of the CoA esters were stored at −20°C.

### Strains and Culture Conditions

Cells of *M. sedula* TH2^T^ (DSM 5348) were grown autotrophically at 75°C modified Allen medium, pH 2.0 (Huber and Prangishvili, 2006) in a 100-l fermenter under gassing with a mixture of 19% CO_2_, 3% O_2_, and 78% H_2_ (0.5 l/min) (generation time, 8 h) (Huber et al., 1989, 1992). Cells were frozen in liquid nitrogen and stored at −80°C until use. *Escherichia coli* strain DH5α and *E. coli* strain Rosetta 2 (DE3) (Merck, Germany) were grown at 37°C in lysogeny broth (LB) medium. Antibiotics were added to the cultures to a final concentration of 100 μg ampicillin ml^–1^ and 34 μg chloramphenicol ml^–1^.

### Preparation of Cell Extracts

Frozen cells were suspended in a double volume of 20 mM Tris–HCl (pH 7.8) containing 0.1 mg ml^–1^ DNase I. The cell suspensions were passed twice through a chilled French pressure cell at 137 MPa, and the cell lysates were centrifuged for 1 h (100,000 × *g*; 4°C). The supernatant (cell extract) was used for protein purification or enzyme assays immediately.

### Cloning of *M. sedula* Genes in *E. coli*

Standard protocols were used for purification, cloning, transformation, and amplification of DNA (Ausubel et al., 1987). Plasmid DNA was isolated with the Monarch Plasmid Miniprep Kit (NEB). Primers and restriction enzymes used for the cloning of *M. sedula* genes are listed in Supplementary Table S1. The genes were amplified using Q5 polymerase (NEB). PCR conditions were as follows: 35 cycles of 10-s denaturation at 98°C, 30-s primer annealing at 60°C and 30-s elongation at 72°C. The PCR products were treated with the corresponding restrictases (Supplementary Table S1), and the genes were separately ligated into the expression vector pET16b containing a sequence encoding an N-terminal His_10_-tag. The plasmids were transformed into *E. coli* DH5α for amplification, followed by their purification and sequencing.

### Heterologous Expression of *M. sedula* Genes in *E. coli*

The amplified expression vectors were used to transform *E. coli* Rosetta 2 (DE3). The cells were grown at 37°C in LB medium with ampicillin and chloramphenicol. Expression was induced at an optical density (OD_578 nm_) of 0.6 with 1 mM isopropyl-ß-D-thiogalactopyranoside (IPTG), and the temperature was lowered to 20°C. The cells were harvested after additional growth for 4 h and stored at −20°C until use.

### Purification of Recombinant Proteins

Recombinant crotonyl-CoA hydratase/(*S*)-3-hydroxybutyryl-CoA dehydrogenase Msed_0399 was produced untagged. The protein was purified as described previously (Ramos-Vera et al., 2011). The heterologously produced His_10_-tagged (*S*)-3-hydroxybutyryl-CoA dehydrogenases Msed_1423 and Msed_0389 as well as crotonyl-CoA hydratases Msed_0336 and Msed_0384 were purified using affinity chromatography. The corresponding cell extracts were applied at a flow rate of 0.5 ml min^–1^ to a 1-ml Protino Ni-NTA column (Macherey-Nagel) that had been equilibrated with 20 mM Tris–HCl containing 150 mM KCl (pH 7.8 for Msed_1423 and Msed_0389, pH 7.6 for Msed_0384, and pH 8.0 for Msed_0336). The column was washed with the same buffer containing 50 mM imidazole at a flow rate of 0.5 ml min^–1^ to elute unwanted protein. The recombinant enzymes were eluted with the same buffer containing 500 mM imidazole. The enzymes were concentrated using 10K Vivaspin Turbo 4 (Sartorius, Göttingen, Germany) and stored at −20°C with 50% glycerol. The identity of the purified recombinant proteins was confirmed using gel digestion by trypsin followed by LC-MS/MS (Supplementary Table S2).

### Enzyme Assays

The 3-hydroxybutyryl-CoA dehydratase and 3-hydroxypropionyl-CoA dehydratase activities were measured at 42°C. All other enzyme activities were measured at 65°C.

#### Crotonyl-CoA Hydratase

Crotonyl-CoA hydratase activity was measured using ultra-high-performance liquid chromatography (UHPLC) by monitoring crotonyl-CoA-dependent formation of 3-hydroxybutyryl-CoA. The reaction mixture (20 μl) contained 100 mM Tris–HCl (pH 7.8), 1 mM crotonyl-CoA, and purified enzyme. The reaction was stopped after 1 min by the addition of 20 μl of 1 M HCl/10% acetonitrile. Protein was removed by centrifugation, and the samples were analyzed by UHPLC using a reverse-phase (RP) C_18_ column. For *K*_m_ determination, the concentration of crotonyl-CoA was varied (0.01–5 mM). For the measurement of crotonyl-CoA hydratase activity of Msed_0399, 100 mM Tris–HCl (pH 9) was used.

#### 3-Hydroxybutyryl-CoA Dehydratase

3-Hydroxybutyryl-CoA dehydratase activity was measured spectrophotometrically by coupling the reaction to crotonyl-CoA carboxylase/reductase (Ccr) from *Rhodobacter sphaeroides* which reductively carboxylates crotonyl-CoA and acryloyl-CoA to ethylmalonyl-CoA and methylmalonyl-CoA, respectively (Erb et al., 2009). The reaction mixture (20 μl) contained 100 mM Tris–HCl (pH 7.8), 1 mM 3-hydroxybutyryl-CoA, 1 mM NADPH, 0.1 mg ml^–1^ Ccr, 50 mM NaHCO_3_, and purified enzyme. The reaction was stopped as described above, and the samples were analyzed by RP-C_18_ UHPLC. For *K*_m_ determination, the concentration of 3-hydroxybutyryl-CoA was varied (0.01–4 mM).

#### 3-Hydroxypropionyl-CoA Dehydratase

3-Hydroxypropionyl-CoA dehydratase was measured spectrophotometrically as 3-hydroxypropionyl-CoA-dependent acryloyl-CoA formation by coupling the reaction to Ccr. The reaction mixture (20 μl) contained 100 mM MOPS/KOH (pH 7.0), 1 mM 3-hydroxypropionyl-CoA, 1 mM NADPH, 0.1 mg ml^–1^ Ccr, 50 mM NaHCO_3_, and purified enzyme. The reaction was stopped as described above, and the samples were analyzed by RP-C_18_ UHPLC. For *K*_m_ determination, the concentration of 3-hydroxypropionyl-CoA was varied (0.01–1.5 mM).

#### Acryloyl-CoA Hydratase

Acryloyl-CoA hydratase activity was measured using UHPLC by monitoring 3-hydroxypropionyl-CoA formation from acryloyl-CoA. The reaction mixture (20 μl) contained 100 mM Tris–HCl (pH 7.8), 1 mM acryloyl-CoA, and purified enzyme. The reaction was stopped as described above, and the samples were analyzed by RP-C_18_ UHPLC. For *K*_m_ determination, the concentration of acryloyl-CoA was varied (0.02–4 mM).

#### Methacrylyl-CoA Hydratase

Methacrylyl-CoA hydratase activity was measured using UHPLC by monitoring 3-hydroxy-2-methylpropionyl-CoA formation from methacrylyl-CoA. The reaction mixture (20 μl) contained 100 mM Tris–HCl (pH 7.8), 1 mM methacrylyl-CoA, and purified enzyme. The reaction was stopped as described above, and the samples were analyzed by RP-C_18_ UHPLC. For *K*_m_ determination, the concentration of methacrylyl-CoA was varied (0.01–5 mM).

#### (*E*)-2-Octenoyl-CoA Hydratase

(*E*)-2-octenoyl-CoA hydratase activity was measured with UHPLC by monitoring 3-hydroxyoctanoyl-CoA formation from (*E*)-2-octenoyl-CoA. The reaction mixture (20 μl) contained 100 mM Tris–HCl (pH 7.8), 1 mM (*E*)-2-octenoyl-CoA, and purified enzyme. The reaction was stopped as described above, and the samples were analyzed by RP-C_18_ UHPLC. For *K*_m_ determination, the concentration of (*E*)-2-octenoyl-CoA was varied (0.01–2 mM).

### Analytical UHPLC

CoA and CoA-esters were detected with Agilent 1290 Infinity II UHPLC using a reversed-phase C18 column (Agilent InfinityLab Poroshell 120 EC-C18 1.9 μm 2.1 × 50 mm column). The following acetonitrile gradient in 10 mM potassium phosphate buffer (pH 7), with a flow rate of 0.55 ml min^–1^, was used: from 2 to 8% at 0–2.66 min; from 8 to 30% at 2.66–3.33 min; from 30 to 2% at 3.33–3.68 min; 2% at 3.68–5 min. Retention times were: 2-methylmalonyl-CoA, 0.9 min; 3-hydroxypropionyl-CoA, 1.4 min; acetyl-CoA, 1.6 min; 3-hydroxybutyryl-CoA, 1.8 min; 3-hydroxy-2-methylpropionyl-CoA, 2.0 min; acryloyl-CoA, 2.2 min; propionyl-CoA, 2.4 min; crotonyl-CoA, 2.6 min; methacrylyl-CoA, 2.9 min. For the conversions with (*E*)-2-octenoyl-CoA, the following acetonitrile gradient in 10 mM potassium phosphate buffer (pH 6.8), with a flow rate of 0.55 ml min^–1^, was used: from 2 to 30% at 0–3.33 min; from 30 to 2% at 3.33–3.68 min; 2% at 3.68–5 min. Retention times were: 3-hydroxyoctanoyl-CoA, 2.3 min; 3-oxo-octanoyl-CoA, 2.4 min; (*E*)-2-octenoyl-CoA, 3.2 min. Reaction products and standard compounds were detected by UV absorbance at 260 nm with 1290 Infinity II diode array detector (Agilent), and the amount of product was calculated from the relative peak area. The identification of the CoA esters was based on co-chromatography with standards and analysis of the UV spectra of the products.

### Other Methods

Apparent *K*_m_ and *V*_max_ values were calculated using GraphPad Prism5 software. Protein concentration was determined using the Bradford method (Bradford, 1976) with bovine serum albumin as a standard. DNA sequence determination was performed by Eurofins (Ebersberg, Germany). Sodium dodecyl sulfate-polyacrylamide gel electrophoresis (SDS-PAGE) (12.5%) was performed as described by Laemmli (1970). An unstained protein MW marker (Thermo Scientific, 14.4-116 kDa) and a prestained protein marker (Cell Signaling, 11-190 kDa) were used as molecular mass standard. Proteins were visualized by Coomassie blue staining (Zehr et al., 1989). Protein identification was performed at the IZKF Core Unit Proteomics Münster based on tryptic in-gel digestion and mass spectrometric analysis using Synapt G2 Si coupled to M-Class (Waters Corp.). Query sequences for the database searches were obtained from the National Center for Biotechnology Information (NCBI) data base. The BLAST searches were performed via NCBI BLAST server^1^ (Altschul et al., 1990).

## Results

### Catalytic Properties of Bifunctional Crotonyl-CoA Hydratase/(*S*)-3-Hydroxybutyryl-CoA Dehydrogenase Msed_0399

Msed_0399 is a bifunctional crotonyl-CoA hydratase/(*S*)-3-hydroxybutyryl-CoA dehydrogenase consisting of two domains, a C-terminal enoyl-CoA hydratase domain (∼20 kDa) and an N-terminal dehydrogenase domain (∼40 kDa) (Ramos-Vera et al., 2011). This enzyme is widely distributed among Archaea and has been predicted to be present in the common ancestor of the TACK superphylum, i.e., it was already present in the common ancestor of Cren- and Thaumarchaeota (Williams et al., 2017). The enzyme was purified formerly from autotrophically grown *M. sedula* cells, following the reduction of NAD with crotonyl-CoA, i.e., the presence of both crotonase and dehydrogenase reactions simultaneously (Ramos-Vera et al., 2011). The identified *msed_0399* was cloned and heterologously produced, and the recombinant enzyme was characterized (Ramos-Vera et al., 2011; Hawkins et al., 2014). However, the characterization was preliminary, as the activity was measured only as crotonyl-CoA or (*S*)-3-hydroxybutyryl-CoA-dependent NAD reduction (Ramos-Vera et al., 2011; Hawkins et al., 2014). The measured hydratase activity in the coupled assay was necessarily limited by the level of the much lower dehydrogenase activity.

In order to fill this gap, we heterologously produced and purified the bifunctional protein Msed_0399, and characterized the enzyme by measuring dehydrogenase and crotonase reactions separately. In hydratase reaction, Msed_0399 was promiscuous and could hydrate not only crotonyl-CoA, but also (*E*)-2-octenoyl-CoA and acrylyl-CoA (Table 2). Similarly, it acted as dehydrogenase with both C_4_ and C_8_ (*S*)-3-hydroxyacyl-CoAs (Table 2). Interestingly, the enzyme was most active with octenoyl-CoA, thus suggesting that Msed_0399 may participate in β-oxidation, in addition to autotrophic CO_2_ fixation. Importantly, Msed_0399 is the only 3-hydroxyacyl-CoA dehydrogenase that was active with C_8_ (3-hydroxy)acyl-CoAs (Tables 2, 3), further suggesting its participation in heterotrophic metabolism.

**TABLE 2:** Catalytic properties of crotonyl-CoA hydratase/(*S*)-3-hydroxybutyryl-CoA dehydrogenase Msed_0399 and the corresponding activities in *M. sedula* cell extract.

Substrate^a^	Msed_0399				*M. sedula* (cell extract)		
	*V*_max_ (U mg^–1^ protein)		*K*_m_ (mM)	*k*_cat_*/K_m_*^a^	*V*_max_ (U mg^–1^ protein)		*K*_m_ (mM)
	Measured (65°C)	Extrapolated to 75°C			Measured (65°C)	Extrapolated to 75°C	
Crotonyl-CoA hydratase	263 ± 7	526 ± 14	0.97 ± 0.07	640	16 ± 4	32 ± 8	ND
(*S*)-3-Hydroxybutyryl-CoA dehydratase^b^	25 ± 1^b^	246 ± 10	0.86 ± 0.12	338	2 ± 1^b^	20 ± 10	ND
Acrylyl-CoA hydratase	186 ± 11	372 ± 22	1.1 ± 0.20	399	132 ± 4	264 ± 8	ND
3-Hydroxypropionyl-CoA dehydratase^b^	0.49 ± 0.04^b^	4.8 ± 0.39	0.06 ± 0.02	94	1 ± 0.2^b^	10 ± 2	ND
Methacrylyl-CoA hydratase	1.3 ± 0.1	2.6 ± 0.2	0.79 ± 0.12	4	2 ± 0.1	4 ± 0.2	ND
(*E*)-2-Octenoyl-CoA hydratase	76 ± 2	152 ± 4	0.08 ± 0.01	2,243	12 ± 4	24 ± 8	ND
Crotonyl-CoA hydratase/(*S*)-3-hydroxybutyryl-CoA dehydrogenase (NAD)^c^	35 ± 1	70 ± 2	0.30 ± 0.03^d^	275	0.8 ± 0.06	1.6 ± 0.1	0.07 ± 0.02
(*S*)-3-hydroxybutyryl-CoA dehydrogenase (NAD)	38 ± 1	76 ± 2	0.12 ± 0.01^d^	748	1.3 ± 0.05	2.6 ± 0.1	0.04 ± 0.01
(*E*)-2-octenoyl-CoA hydratase/3-hydroxyoctanoyl-CoA dehydrogenase (NAD)^c^	5.2 ± 0.4	10.4 ± 0.8	0.02 ± 0.01	614	0.75 ± 0.07	1.5 ± 0.1	0.05 ± 0.02

*The V_max_/specific activity values are normalized to 75°C based on the assumption that a 10°C rise in temperature doubles the reaction rate. ND, not determined. ^a^k_cat_ was calculated in s^–1^ mM^–1^ for the activities at 75°C. ^b^Activity was measured at 42°C. ^c^The reaction was measured with the corresponding enoyl-CoA as a substrate. ^d^The following K_m_ values were detected previously: crotonyl-CoA, 0.3 and 0.07 mM, (S)-3-hydroxybutyryl-CoA, 0.2 and 0.06 mM (Ramos-Vera et al., 2011; Hawkins et al., 2014).*

**TABLE 3:** Catalytic properties of (*S*)-3-hydroxybutyryl-CoA dehydrogenases Msed_1423 and Msed_0389.

Substrate^a^	Msed_1423				Msed_0389			
	*V*_max_ (U mg^–1^ protein)		*K*_m_ (mM)	*k*_cat_/*K*_m_^a^	*V*_max_ (U mg^–1^ protein)		*K*_m_ (mM)	*k*_cat_/*K*_m_^a^
	Measured (65°C)	Extrapolated to 75°C			Measured (65°C)	Extrapolated to 75°C		
(*S*)-3-Hydroxybutyryl-CoA (with 0.5 mM NAD)	48 ± 3	96 ± 6	0.005 ± 0.002	14,947	4.8 ± 0.2	9.6 ± 0.4	0.0026 ± 0.001	2,476
NAD	41 ± 1	82 ± 2	0.03 ± 0.003	2,128	4.8 ± 0.1	9.6 ± 0.2	0.012 ± 0.002	536
NADP	43 ± 1	86 ± 2	5.1 ± 0.5	13	2.9 ± 0.2	5.8 ± 0.4	1.5 ± 0.3	3

*The V_max_ values are normalized to 75°C based on the assumption that a 10°C rise in temperature doubles the reaction rate. No activity was detected with (S)-3-hydroxyoctanoyl-CoA (with 0.5 mM NAD; detection limit ≤0.2 U mg^–1^ for Msed_1423 and ≤0.1 U mg^–1^ for Msed_0389). ^a^k_cat_ was calculated in s^–1^ mM^–1^ for the activities at 75°C.*

### Crotonyl-CoA Hydratase and (*S*)-3-Hydroxybutyryl-CoA Dehydrogenase Activities in *M. sedula* Cell Extract

The apparent *K*_m_ value of Msed_0399 for crotonyl-CoA in the crotonase/(*S*)-3-hydroxybutyryl-CoA dehydrogenase reaction was four times higher than the corresponding value in *M. sedula* cell extract (Table 2) suggesting the presence of additional crotonase(s). Furthermore, the apparent *K*_m_ value of Msed_0399 for (*S*)-3-hydroxybutyryl-CoA in the dehydrogenase reaction was three times higher than the corresponding value in *M. sedula* cell extract (Table 2). These data suggest that Msed_0399 may not be the only enzyme responsible for crotonyl-CoA hydratase and (*S*)-3-hydroxybutyryl-CoA dehydrogenase activity in autotrophically grown *M. sedula*. Therefore, we cloned and expressed other candidate genes for these enzymes (Table 1) in *E. coli* and characterized the catalytic activities of the resulting enzymes.

### 3-Hydroxybutyryl-CoA Dehydrogenases Msed_0389 and Msed_1423

**FIGURE 2:**
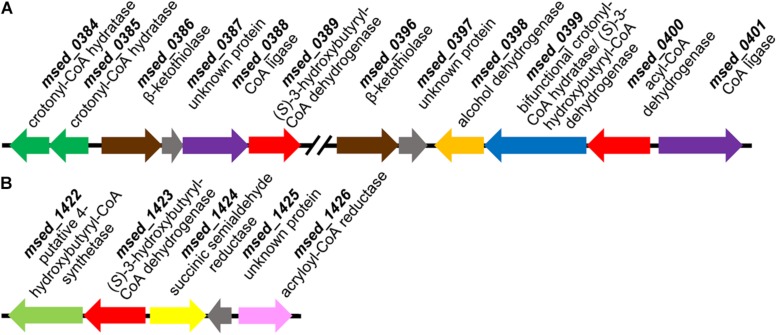
Gene clusters encoding (*S*)-3-hydroxybutyryl-CoA dehydrogenase Msed_0389 **(A)** and Msed_1423 **(B)**. Genes: **(A)** *msed_0384*, *msed_0385*, crotonyl-CoA hydratases; *msed_0386*, *msed_0396*, β-ketothiolases; *msed_0387*, *msed_0397*, unknown proteins; *msed_0388*, CoA ligase; *msed_0389*, (*S*)-3-hydroxybutyryl-CoA dehydrogenase; *msed_0398*, alcohol dehydrogenase; *msed_0399*, bifunctional crotonyl-CoA hydratase/(*S*)-3-hydroxybutyryl-CoA dehydrogenase; *msed_0400*, acyl-CoA dehydrogenase; *msed_0401*, CoA ligase. **(B)** *msed_1422*, putative 4-hydroxybutyryl-CoA synthetase; *msed_1423*, (*S*)-3-hydroxybutyryl-CoA dehydrogenase; *msed_1424*, succinic semialdehyde reductase; *msed_1425*, unknown protein; *msed_1426*, acryloyl-CoA reductase.

The purified recombinant Msed_0389 and Msed_1423 catalyzed the NAD-dependent oxidation of (*S*)-3-hydroxybutyryl-CoA to acetoacetyl-CoA and were highly specific for their substrate (*K*_m_ 2.6 and 5 μM, respectively; Table 3). Both enzymes were not active with (*S*)-3-hydroxyoctanoyl-CoA. Their *k*_cat_/*K*_m_ values were 3- and 20-fold higher than the corresponding value of Msed_0399 in (*S*)-3-hydroxybutyryl-CoA dehydrogenase reaction (Table 3). Interestingly, both these genes were shown to be up-regulated in auto- and mixotrophically grown cell (see Table 1; Auernik and Kelly, 2010; Ai et al., 2019). The Msed_0389 gene clustered together with various β-oxidation genes in the *M. sedula* genome. In contrast, *msed_1423* clustered with several genes encoding specific enzymes of the HP/HB cycle, suggesting its involvement in autotrophic CO_2_ fixation (Figure 2).

### Crotonyl-CoA Hydratases Msed_0336 and Msed_0384

The purified recombinant Msed_0336 and Msed_0384 catalyzed the hydration of crotonyl-CoA to (*S*)-3-hydroxybutyryl-CoA (Table 4); the *k*_cat_/*K*_m_ values of both enzymes for the reverse reaction were ∼2.5 times higher. Both enzymes were also active with (*E*)-2-octenoyl-CoA and, to a lesser extent, with acrylyl-CoA and methacrylyl-CoA. The *V*_max_ values of these enzymes for crotonyl-CoA hydration were lower than the *V*_max_ of Msed_0399; yet their apparent crotonyl-CoA *K*_m_ values were also much lower, resulting in a catalytic constant *k*_cat_/*K*_m_ similar to that of Msed_0399. The clustering of *msed_0384* with β-oxidation genes conforms with its higher *k*_cat_/*K*_m_ value for octenoyl-CoA. Nevertheless, it is Msed_0399 which has the highest *k*_cat_/*K*_m_ value for this substrate. In the published transcriptomic analyses, Msed_0384 was shown to be down-regulated in auto- and mixotrophically grown cell, while Msed_0336 showed no significant regulation in these cells (see Table 1; Auernik and Kelly, 2010; Ai et al., 2019).

**TABLE 4:** Catalytic properties of crotonyl-CoA hydratases Msed_0336 and Msed_0384.

Substrate^a^	Msed_0336				Msed_0384			
	*V*_max_ (U mg^–1^ protein)		*K*_m_ (mM)	*k*_cat_/*K*_m_^a^	*V*_max_ (U mg^–1^ protein)		*K*_m_ (mM)	*k*_cat_/*K*_m_^a^
	Measured (65°C)	Extrapolated to 75°C			Measured (65°C)	Extrapolated to 75°C		
Crotonyl-CoA	26 ± 1	52 ± 2	0.08 ± 0.01	317	227 ± 7	454 ± 14	0.22 ± 0.02	1,043
(*S*)-3-hydroxybutyryl-CoA^b^	12 ± 0.8^*c*^	119 ± 8	0.07 ± 0.02	830	25 ± 1^*c*^	242 ± 12	0.05 ± 0.01	2,445
Acrylyl-CoA	9 ± 0.2	18 ± 0.4	0.07 ± 0.005	126	25 ± 1	50 ± 2	0.18 ± 0.03	140
3-Hydroxypropionyl-CoA	0.4^c,d^	4^d^	NA	NA	0.4^c,d^	4^d^	NA	NA
Methacrylyl-CoA	17 ± 1	34 ± 2	0.29 ± 0.05	57	0.7 ± 0.1	1.4 ± 0.2	0.07 ± 0.03	10
(*E*)-2-octenoyl-CoA	12 ± 0.4	24 ± 0.8	0.09 ± 0.01	130	123 ± 5	246 ± 10	0.5 ± 0.06	249

*The V_max_ values are normalized to 75°C based on the assumption that a 10°C rise in temperature doubles the reaction rate. NA, not applicable. ^a^k_cat_ was calculated in s^–1^ mM^–1^ for the activities at 75°C. ^b^No activity was detected with (R)-3-hydroxybutyryl-CoA (detection limit ≤0.3 U mg^–1^ Msed_0336, ≤1 U mg^–1^ Msed_0384). ^c^Activity was measured at 42°C. ^d^Specific activity.*

### Distribution of Crotonase and (*S*)-3-Hydroxybutyryl-CoA Dehydrogenase Genes in Sulfolobales

The genes of the HP/HB cycle catalyzing reactions from acetyl-CoA to crotonyl-CoA are present in all fully sequenced Sulfolobales genomes (Supplementary Table S3), indicating that they have the potential to grow autotrophically. The only exception was *Sulfodiicoccus acidiphilus* that does not possess most of the key genes of the HP/HB cycle. This species is only distantly related to other Sulfolobales and is not capable to grow autotrophically (Sakai and Kurosawa, 2017, 2019). On the contrary, most of the other Sulfolobales are autotrophs, with the exception of *Sulfolobus acidocaldarius* and *Saccharolobus solfataricus* that nevertheless encode all key genes of the HP/HB cycle in their genomes (Supplementary Table S3).

Homolog(s) of bifunctional crotonyl-CoA hydratase/(*S*)-3-hydroxybutyryl-CoA dehydrogenase Msed_0399 genes were found in 75% of the genomes, whereas four autotrophic species (*Acidianus ambivalens*, *Acidianus hospitalis*, *Acidianus sulfidivorans*, and *Stygiolobus azoricus*) do not possess the corresponding gene (Table 5). Notably, the functioning of the HP/HB cycle was experimentally shown for *S. azoricus* (Berg et al., 2010b), indicating that the presence of the bifunctional protein is not essential for autotrophic CO_2_ fixation. The only two genes that are (almost) universally distributed in Sulfolobales are the crotonase Msed_0566 gene and the dehydrogenase Msed_1423 gene (Table 5 and Supplementary Table S3). They are absent only in the genome of *S. acidocaldarius*.

**TABLE 5:** Distribution of genes encoding crotonyl-CoA hydratase (CCH) and (*S*)-3-hydroxybutyryl-CoA dehydrogenase (HBDH) in the genomes of Sulfolobales.

	CCH/HBDH	CCH				HBDH	
	Msed_0399	Msed_0384	Msed_0385	Msed_0336	Msed_0566	Msed_1423	Msed_0389
*Acidianus ambivalens* LEI 10	–	–	–	–	50%	71%	–
*Acidianus brierleyi* DSM 1651	58%	66%	55%	52%	52%	72%	48%
*Acidianus hospitalis* W1	–	–	–	–	49%	71%	–
*Acidianus manzaensis* YN-25	52%	72%	60%	58%	47%	71%	42%
*Acidianus sulfidivorans* JP7	±49%	71%	62%	57%	47%	72%	54%
*Candidatus* Acidianus copahuensis ALE1	49%	72%	59%	46%	47%	72%	54%
*Metallosphaera cuprina* Ar-4	71%	78%	80%	–	76%	82%	71%
*Metallosphaera hakonensis* JCM 8857	82%	84%	81%	71%	81%	87%	79%
*Metallosphaera prunae* Ron 12/II	100%	100%	100%	100%	100%	100%	100%
*Metallosphaera yellowstonensis* MK1	64%	74%	72%	59%	75%	77%	69
*Saccharolobus solfataricus* P2	53%	68%	53%	58%	46%	67%	45%^c^
*Stygiolobus azoricus* FC6	–	–	–	–	46%	69%	–
*Sulfolobus acidocaldarius* DSM 639	53%^b^	70%	50%	–	–	47%^c^	–
*Sulfolobus islandicus* L.S.2.15	53%^d^	66%	51%	58%	44%	68%	45%^c^
*Sulfolobus* sp. A20	53%^d^	65%	53%	55%	40%	71%	46%^c^
*Sulfurisphaera tokodaii* 7	54%	74%	64%	51%	44%	69%	57%
*Sulfodiicoccus acidiphilus* HS-1	38%	68%	54%	–	36%	59%	59%

*Only fully sequenced genomes were considered. The values are given in % identity to the corresponding M. sedula proteins. For the full data set, see Supplementary Table S3. ^a^Only dehydrogenase domain of the bifunctional fusion protein is present. ^b^Four homologous fusion proteins in the genome. ^c^Protein homologous to the dehydrogenase domain of Msed_0399 rather than to Msed_1423. ^d^Two homologous fusion proteins in the genome.*

## Discussion

Genome analysis shows that *M. sedula* and most other Sulfolobales have multiple homologs of genes responsible for the conversion of crotonyl-CoA into acetyl-CoA, and our data indeed suggest that crotonyl-CoA hydratase and (*S*)-3-hydroxybutyryl-CoA dehydrogenase reactions may be catalyzed by several proteins *in vivo*. It is probably also true for the β-ketothiolase reaction cleaving acetoacetyl-CoA into two molecules of acetyl-CoA, as at least one of the homologous genes (*msed_0386*) is up-regulated under mixo- and autotrophic growth conditions, in addition to the characterized β-ketothiolase Msed_0656 gene (Table 1; Auernik and Kelly, 2010; Hawkins et al., 2013a; Ai et al., 2019). The studied crotonases are homologous to 3-hydroxypropionyl-CoA dehydratase Msed_2001 and possess low 3-hydroxypropionyl-CoA dehydratase activity (Table 2), thus probably also contributing to 3-hydroxypropionyl-CoA dehydratase activity in the cells. 3-Hydroxypropionyl-CoA dehydratase, in turn, has crotonase activity (Teufel et al., 2009). Although analysis of deletion mutants would be very helpful to clarify the role of individual proteins in autotrophic CO_2_ fixation, genetic system is unfortunately not available for autotrophic Sulfolobales.

In contrast to the current understanding, our data show that the bifunctional crotonyl-CoA hydratase/(*S*)-3-hydroxybutyryl-CoA dehydrogenase is not the main enzyme that catalyzes these reactions in *M. sedula.* This is corroborated by the fact that its gene is absent in many other autotrophic Sulfolobales. In fact, *msed_0399* is located in the cluster with the genes homologous to various β-oxidation genes (Figure 2A) and may thus be primarily involved in, e.g., fatty acid metabolism rather than CO_2_ fixation (Table 2). Although the growth of *M. sedula* on fatty acids has not been shown, some Sulfolobales are capable to use these compounds as a sole carbon source (Wang et al., 2019). Furthermore, reversibility of β-oxidation enzymes in Archaea and their involvement in fatty acid synthesis was proposed (Dibrova et al., 2014).

Interestingly and in contrast to Sulfolobales, the homologous bifunctional protein is the only enzyme capable to covert crotonyl-CoA into acetoacetyl-CoA in autotrophic Desulfurococcales (*Ignicoccus hospitalis*) and Thermoproteales (*Pyrobaculum neutrophilus*) that use the dicarboxylate/4-hydroxybutyrate cycle for CO_2_ fixation (Huber et al., 2008; Ramos-Vera et al., 2009, 2011). This cycle differs from the HP/HB cycle in the carboxylation reactions (ferredoxin-dependent pyruvate synthase and phosphoenolpyruvate carboxylase) but shares the regeneration part, i.e., the conversion of succinyl-CoA into acetyl-CoA that includes crotonase and (*S*)-3-hydroxybutyryl-CoA dehydrogenase reactions. The anaerobic dicarboxylate/4-hydroxybutyrate cycle is probably the ancestral CO_2_ fixation pathway in Crenarchaeota, and the presence of the bifunctional protein widely distributed among Archaea seems to be an ancestral feature for the pathway.

Nevertheless, even if several enzymes catalyze one and the same reactions in *M. sedula*, our biochemical data (Table 3) and the results of our gene distribution analysis (Table 5) together with the published transcriptomic data (see Table 1) show that Msed_1423 is the main (*S*)-3-hydroxybutyryl-CoA dehydrogenase in the HP/HB cycle in Sulfolobales. This protein is clustered together with two specific enzymes of the HP/HB cycle, succinic semialdehyde reductase Msed_1424 and acryloyl-CoA reductase Msed_1426 (Figure 2B), further supporting its involvement in autotrophic CO_2_ fixation. The only species that does not possess the corresponding gene, *S. acidocaldarius*, is not capable to grow autotrophically (Zeldes et al., 2019).

The crotonase homolog, Msed_0566, was the only monofunctional crotonase (besides the promiscuous 3-hydroxypropionyl-CoA dehydratase) that was present in all Sulfolobales except for the heterotrophic *S. acidocaldarius* (Table 5). Unfortunately, all our attempts to heterologously express *msed_0566* (as well as *msed_0385*) were not successful and resulted in insoluble protein. It is to be shown whether this protein is the primary crotonyl-CoA hydratase in *M. sedula* and especially in *S. azoricus*, *A. ambivalens*, and *A. hospitalis* (Table 5), or this reaction is also catalyzed by a promiscuous 3-hydroxypropionyl-CoA dehydratase, as was shown for the HP/HB cycle in Thaumarchaeota (Könneke et al., 2014).

The inability of *S. acidocaldarius* to grow autotrophically is well-known (Berg et al., 2010b; Zeldes et al., 2019). It has recently been suggested that the obligate heterotrophy of this organism is rooted rather in gene regulation than in the biochemical capacity (Zeldes et al., 2019). Our data suggest that this inability is likely due to the absence of the Msed_1423 (and possibly Msed_0566) homologs in this species.

Comparison of the activities of the characterized (*S*)-3-hydroxybutyryl-CoA dehydrogenase Msed_1423 and crotonase Msed_0384 with the activities of these reactions in cell extract of *M. sedula* leads to the values 1.7 and 7.1% of total cellular protein, respectively. The last value was calculated based on the activity of crotonase found in our work (Table 2), which is twice higher than the previously measured activity (Berg et al., 2007; see Table 1). With these corrected values, the estimated abundance of the HP/HB cycle enzymes is ∼47% of total cellular protein, i.e., 11% less than was calculated in Table 1. This value may further be corrected (reduced?) after the characterization of the further crotonase homologs in *M. sedula*.

The studied enzymes were promiscuous, differing in their kinetic properties. Their simultaneous presence not only increases the activity of particular reactions, but also allows fast and efficient fine-tuning of metabolism and adaptation to different growth conditions, varying metabolite concentrations and growth rates. This strategy may be especially advantageous for metabolically versatile organisms that are not limited in energy source.

The HP/HB cycle is a perspective route for the conversion of CO_2_ into liquid fuels and industrial chemicals and can be engineered for the production of value-added compounds like 3-hydroxypropionate or *n*-butanol (Hawkins et al., 2011, 2013b; Keller et al., 2013; Fuchs and Berg, 2014; Loder et al., 2016). The identification of highly specific thermophilic enzymes involved in this pathway expands opportunities for the application of the pathway. Simultaneous usage of several isoenzymes differing in their kinetic properties is an interesting concept for biotechnology that may allow stable production under varying conditions.

## Data Availability Statement

The datasets generated for this study are available on request to the corresponding author.

## Author Contributions

IB designed the experiments. LL and HH performed the experiments. LL, HH, and IB analyzed the data. LL and IB wrote the manuscript. All authors read and approved the final manuscript.

## Conflict of Interest

The authors declare that the research was conducted in the absence of any commercial or financial relationships that could be construed as a potential conflict of interest.

## Footnotes

1 http://www.ncbi.nlm.nih.gov/BLAST/

## References

[B1] Ai C. Yan Z. Chai H. Gu T. Wang J. Chai L. (2019). Increased chalcopyrite bioleaching capabilities of extremely thermoacidophilic *Metallosphaera sedula* inocula by mixotrophic propagation. *J. Ind. Microbiol. Biotechnol.* 46 1113–1127. 10.1007/s10295-019-02193-3 31165968

[B2] Alber B. E. Kung J. W. Fuchs G. (2008). 3-Hydroxypropionyl-coenzyme A synthetase from *Metallosphaera sedula*, an enzyme involved in autotrophic CO_2_ fixation. *J. Bacteriol.* 190 1383–1389. 10.1128/JB.01593-07 18165310 PMC2238213

[B3] Altschul S. F. Gish W. Miller W. Myers E. W. Lipman D. J. (1990). Basic local alignment search tool. *J. Mol. Biol.* 215 403–410. 2231712 10.1016/S0022-2836(05)80360-2

[B4] Auernik K. S. Kelly R. M. (2010). Physiological versatility of the extremely thermoacidophilic archaeon *Metallosphaera sedula* supported by transcriptomic analysis of heterotrophic, autotrophic, and mixotrophic growth. *Appl. Environ. Microbiol.* 76 931–935. 10.1128/AEM.01336-09 20008169 PMC2813022

[B5] Ausubel F. M. Brent R. Kingston R. E. Moore D. D. Seidman J. G. Smith J. A. (1987). *Current Protocols in Molecular Biology.* New York, NY: John Wiley and Sons.

[B6] Berg I. A. (2011). Ecological aspects of the distribution of different autotrophic CO_2_ fixation pathways. *Appl. Environ. Microbiol.* 77 1925–1936. 10.1128/AEM.02473-10 21216907 PMC3067309

[B7] Berg I. A. Kockelkorn D. Buckel W. Fuchs G. (2007). A 3-hydroxypropionate/4-hydroxybutyrate autotrophic carbon dioxide assimilation pathway in Archaea. *Science* 318 1782–1786. 10.1126/science.1149976 18079405

[B8] Berg I. A. Kockelkorn D. Ramos-Vera W. H. Say R. F. Zarzycki J. Hügler M. (2010a). Autotrophic carbon fixation in archaea. *Nat. Rev. Microbiol.* 8 447–460. 10.1038/nrmicro2365 20453874

[B9] Berg I. A. Ramos-Vera W. H. Petri A. Huber H. Fuchs G. (2010b). Study of the distribution of autotrophic CO_2_ fixation cycles in Crenarchaeota. *Microbiology* 156(Pt 1) 256–269. 10.1099/mic.0.034298-0 19850614

[B10] Bradford M. M. (1976). A rapid and sensitive method for the quantification of microgram quantities of protein utilizing the principle of protein-dye binding. *Anal. Biochem.* 72 248–254. 10.1016/0003-2697(76)90527-3 942051

[B11] Buckel W. (2019). Enzymatic reactions involving ketyls: from a chemical curiosity to a general biochemical mechanism. *Biochemistry* 58 5221–5233. 10.1021/acs.biochem.9b00171 30995029

[B12] Crosby H. A. Heiniger E. K. Harwood C. S. Escalante-Semerena J. C. (2010). Reversible N epsilon-lysine acetylation regulates the activity of acyl-CoA synthetases involved in anaerobic benzoate catabolism in *Rhodopseudomonas palustris*. *Mol. Microbiol.* 76 874–888. 10.1111/j.1365-2958.2010.07127.x 20345662 PMC2913386

[B13] Dibrova D. V. Galperin M. Y. Mulkidjanian A. Y. (2014). Phylogenomic reconstruction of archaeal fatty acid metabolism. *Environ. Microbiol.* 16 907–918. 10.1111/1462-2920.12359 24818264 PMC4019937

[B14] Erb T. J. Brecht V. Fuchs G. Müller M. Alber B. E. (2009). Carboxylation mechanism and stereochemistry of crotonyl-CoA carboxylase/reductase, a carboxylating enoyl-thioester reductase. *Proc. Natl. Acad. Sci. U.S.A.* 106 8871–8876. 10.1073/pnas.0903939106 19458256 PMC2689996

[B15] Fuchs G. (2011). Alternative pathways of carbon dioxide fixation: insights into the early evolution of life? *Annu. Rev. Microbiol.* 65 631–658. 10.1146/annurev-micro-090110-102801 21740227

[B16] Fuchs G. Berg I. A. (2014). Unfamiliar metabolic links in the central carbon metabolism. *J. Biotechnol.* 192(Pt B) 314–322. 10.1016/j.jbiotec.2014.02.015 24576434

[B17] Han Y. Hawkins A. S. Adams M. W. Kelly R. M. (2012). Epimerase (Msed_0639) and mutase (Msed_0638 and Msed_2055) convert (*S*)-methylmalonyl-coenzyme A (CoA) to succinyl-CoA in the *Metallosphaera sedula* 3-hydroxypropionate/4-hydroxybutyrate cycle. *Appl. Environ. Microbiol.* 78 6194–6202. 10.1128/AEM.01312-12 22752162 PMC3416614

[B18] Hawkins A. B. Adams M. W. Kelly R. M. (2014). Conversion of 4-hydroxybutyrate to acetyl coenzyme A and its anapleurosis in the *Metallosphaera sedula* 3-hydroxypropionate/4-hydroxybutyrate carbon fixation pathway. *Appl. Environ. Microbiol.* 80 2536–2545. 10.1128/AEM.04146-13 24532060 PMC3993168

[B19] Hawkins A. S. Han Y. Bennett R. K. Adams M. W. Kelly R. M. (2013a). Role of 4-hydroxybutyrate-CoA synthetase in the CO_2_ fixation cycle in thermoacidophilic archaea. *J. Biol. Chem.* 288 4012–4022. 10.1074/jbc.M112.413195 23258541 PMC3567653

[B20] Hawkins A. S. Han Y. Lian H. Loder A. J. Menon A. L. Iwuchukwu I. J. (2011). Extremely thermophilic routes to microbial electrofuels. *ACS Catal.* 1 1043–1050. 10.1021/cs2003017

[B21] Hawkins A. S. McTernan P. M. Lian H. Kelly R. M. Adams M. W. (2013b). Biological conversion of carbon dioxide and hydrogen into liquid fuels and industrial chemicals. *Curr. Opin. Biotechnol.* 24 376–384. 10.1016/j.copbio.2013.02.017 23510698

[B22] Huber G. Drobner E. Huber H. Stetter K. O. (1992). Growth by aerobic oxidation of molecular hydrogen in Archaea—a metabolic property so far unknown for this domain. *Syst. Appl. Microbiol.* 15 502–504. 10.1016/s0723-2020(11)80108-6

[B23] Huber G. Spinnler C. Gambacorta A. Stetter K. O. (1989). *Metallosphaera sedula* gen. and sp. nov. represents a new genus of aerobic, metal mobilizing, thermoacidophilic Archaebacteria. *Syst. Appl. Microbiol.* 12 38–47. 10.1016/s0723-2020(89)80038-4

[B24] Huber H. Gallenberger M. Jahn U. Eylert E. Berg I. A. Kockelkorn D. (2008). A dicarboxylate/4-hydroxybutyrate autotrophic carbon assimilation cycle in the hyperthermophilic Archaeum *Ignicoccus hospitalis*. *Proc. Natl. Acad. Sci. U.S.A.* 105 7851–7856. 10.1073/pnas.0801043105 18511565 PMC2409403

[B25] Huber H. Prangishvili D. (2006). “Sulfolobales,” in *A Handbook on the Biology of Bacteria: Archaea. Bacteria: Firmicutes, Actinomycetes*, 3rd Edn, Vol. 3 eds Dworkin M. Falkow S. Rosenberg E. Schleifer K.-H., (New York, NY: Springer), 23–51. 10.1007/0-387-30743-5_3

[B26] Hügler M. Krieger R. S. Jahn M. Fuchs G. (2003). Characterization of acetyl-CoA/propionyl-CoA carboxylase in *Metallosphaera sedula*. Carboxylating enzyme in the 3-hydroxypropionate cycle for autotrophic carbon fixation. *Eur. J. Biochem.* 270 736–744. 10.1046/j.1432-1033.2003.03434.x 12581213

[B27] Hügler M. Sievert S. M. (2011). Beyond the Calvin cycle: autotrophic carbon fixation in the ocean. *Ann. Rev. Mar. Sci.* 3 261–289. 10.1146/annurev-marine-120709-142712 21329206

[B28] Ishii M. Miyake T. Satoh T. Sugiyama H. Oshima Y. Kodama T. (1996). Autotrophic carbon dioxide fixation in *Acidianus brierleyi*. *Arch. Microbiol.* 166 368–371. 10.1007/s002030050397 9082912

[B29] Keller M. W. Schut G. J. Lipscomb G. L. Menon A. L. Iwuchukwu I. J. Leuko T. T. (2013). Exploiting microbial hyperthermophilicity to produce an industrial chemical, using hydrogen and carbon dioxide. *Proc. Natl. Acad. Sci. U.S.A.* 110 5840–5845. 10.1073/pnas.1222607110 23530213 PMC3625313

[B30] Kockelkorn D. Fuchs G. (2009). Malonic semialdehyde reductase, succinic semialdehyde reductase, and succinyl-coenzyme A reductase from *Metallosphaera sedula*: enzymes of the autotrophic 3-hydroxypropionate/4-hydroxybutyrate cycle in Sulfolobales. *J. Bacteriol.* 191 6352–6362. 10.1128/JB.00794-09 19684143 PMC2753041

[B31] Könneke M. Schubert D. M. Brown P. C. Hügler M. Standfest S. Schwander T. (2014). Ammonia-oxidizing archaea use the most energy-efficient aerobic pathway for CO_2_ fixation. *Proc. Natl. Acad. Sci. U.S.A.* 111 8239–8244. 10.1073/pnas.1402028111 24843170 PMC4050595

[B32] Laemmli U. K. (1970). Cleavage of structural proteins during assembly of the head of bacteriophage T4. *Nature* 227 680–685. 10.1038/227680a0 5432063

[B33] Loder A. J. Han Y. Hawkins A. B. Lian H. Lipscomb G. L. Schut G. J. (2016). Reaction kinetic analysis of the 3-hydroxypropionate/4-hydroxybutyrate CO_2_ fixation cycle in extremely thermoacidophilic archaea. *Metab. Eng.* 38 446–463. 10.1016/j.ymben.2016.10.009 27771364 PMC5433351

[B34] Martins B. M. Dobbek H. Cinkaya I. Buckel W. Messerschmidt A. (2004). Crystal structure of 4-hydroxybutyryl-CoA dehydratase: radical catalysis involving a [4Fe-4S] cluster and flavin. *Proc. Natl. Acad. Sci. U.S.A.* 101 15645–15649. 10.1073/pnas.0403952101 15496473 PMC524839

[B35] Menendez C. Bauer Z. Huber H. Gad’on N. Stetter K. O. Fuchs G. (1999). Presence of acetyl coenzyme A (CoA) carboxylase and propionyl-CoA carboxylase in autotrophic Crenarchaeota and indication for operation of a 3-hydroxypropionate cycle in autotrophic carbon fixation. *J. Bacteriol.* 181 1088–1098. 10.1128/jb.181.4.1088-1098.1999 9973333 PMC93484

[B36] Otte J. Mall A. Schubert D. M. Könneke M. Berg I. A. (2015). Malonic semialdehyde reductase from the archaeon *Nitrosopumilus maritimus* is involved in the autotrophic 3-hydroxypropionate/4-hydroxybutyrate cycle. *Appl. Environ. Microbiol.* 81 1700–1707. 10.1128/AEM.03390-14 25548047 PMC4325172

[B37] Ramos-Vera W. H. Berg I. A. Fuchs G. (2009). Autotrophic carbon dioxide assimilation in Thermoproteales revisited. *J. Bacteriol.* 191 4286–4297. 10.1128/JB.00145-09 19411323 PMC2698501

[B38] Ramos-Vera W. H. Weiss M. Strittmatter E. Kockelkorn D. Fuchs G. (2011). Identification of missing genes and enzymes for autotrophic carbon fixation in Crenarchaeota. *J. Bacteriol.* 193 1201–1211. 10.1128/JB.01156-10 21169482 PMC3067573

[B39] Russell M. J. Martin W. (2004). The rocky roots of the acetyl-CoA pathway. *Trends Biochem. Sci.* 29 358–363. 10.1016/j.tibs.2004.05.007 15236743

[B40] Sakai H. D. Kurosawa N. (2017). *Sulfodiicoccus acidiphilus* gen. nov., sp. nov., a sulfur-inhibited thermoacidophilic archaeon belonging to the order Sulfolobales isolated from a terrestrial acidic hot spring. *Int. J. Syst. Evol. Microbiol.* 67 1880–1886. 10.1099/ijsem.0.001881 28629504

[B41] Sakai H. D. Kurosawa N. (2019). Complete genome sequence of the *Sulfodiicoccus acidiphilus* strain HS-1(T), the first crenarchaeon that lacks polB3, isolated from an acidic hot spring in Ohwaku-dani, Hakone, Japan. *BMC Res. Notes* 12:444. 10.1186/s13104-019-4488-5 31331368 PMC6647314

[B42] Scherf U. Buckel W. (1993). Purification and properties of an iron-sulfur and FAD-containing 4-hydroxybutyryl-CoA dehydratase/vinylacetyl-CoA delta 3-delta 2-isomerase from *Clostridium aminobutyricum*. *Eur. J. Biochem.* 215 421–429. 10.1111/j.1432-1033.1993.tb18049.x 8344309

[B43] Schwander T. Schada von Borzyskowski L. Burgener S. Cortina N. S. Erb T. J. (2016). A synthetic pathway for the fixation of carbon dioxide in vitro. *Science* 354 900–904. 10.1126/science.aah5237 27856910 PMC5892708

[B44] Selmer T. Willanzheimer A. Hetzel M. (2002). Propionate CoA-transferase from *Clostridium propionicum*. Cloning of gene and identification of glutamate 324 at the active site. *Eur. J. Biochem.* 269 372–380. 10.1046/j.0014-2956.2001.02659.x 11784332

[B45] Simon E. J. Shemin D. (1953). The preparation of S-succinyl coenzyme-A. *J. Am. Chem. Soc.* 75 2520 10.1021/ja01106a522

[B46] Stadtman E. R. (1957). Preparation and assay of acyl coenzyme A and other thiol esters; use of hydroxylamine. *Methods Enzymol.* 3 931–941. 10.1016/s0076-6879(57)03481-3

[B47] Teufel R. Kung J. W. Kockelkorn D. Alber B. E. Fuchs G. (2009). 3-hydroxypropionyl-coenzyme A dehydratase and acryloyl-coenzyme A reductase, enzymes of the autotrophic 3-hydroxypropionate/4-hydroxybutyrate cycle in the Sulfolobales. *J. Bacteriol.* 191 4572–4581. 10.1128/JB.00068-09 19429610 PMC2704735

[B48] Wächtershäuser G. (1988). Before enzymes and templates: theory of surface metabolism. *Microbiol. Rev.* 52 452–484. 10.1128/mmbr.52.4.452-484.1988 3070320 PMC373159

[B49] Wang K. Sybers D. Maklad H. R. Lemmens L. Lewyllie C. Zhou X. (2019). A TetR-family transcription factor regulates fatty acid metabolism in the archaeal model organism *Sulfolobus acidocaldarius*. *Nat. Commun.* 10:1542. 10.1038/s41467-019-09479-1 30948713 PMC6449355

[B50] Weiss M. C. Sousa F. L. Mrnjavac N. Neukirchen S. Roettger M. Nelson-Sathi S. (2016). The physiology and habitat of the last universal common ancestor. *Nat. Microbiol.* 1:16116. 10.1038/nmicrobiol.2016.116 27562259

[B51] Williams T. A. Szöllõsi G. J. Spang A. Foster P. G. Heaps S. E. Boussau B. (2017). Integrative modeling of gene and genome evolution roots the archaeal tree of life. *Proc. Natl. Acad. Sci. U.S.A.* 114 E4602–E4611. 10.1073/pnas.1618463114 28533395 PMC5468678

[B52] Zarzycki J. Brecht V. Müller M. Fuchs G. (2009). Identifying the missing steps of the autotrophic 3-hydroxypropionate CO_2_ fixation cycle in *Chloroflexus aurantiacus*. *Proc. Natl. Acad. Sci. U.S.A.* 106 21317–21322. 10.1073/pnas.0908356106 19955419 PMC2795484

[B53] Zehr B. D. Savin T. J. Hall R. E. (1989). A one-step, low-background Coomassie staining procedure for polyacrylamide gels. *Anal. Biochem.* 182 157–159. 10.1016/0003-2697(89)90734-3 2481413

[B54] Zeldes B. M. Loder A. J. Counts J. A. Haque M. Widney K. A. Keller L. M. (2019). Determinants of sulphur chemolithoautotrophy in the extremely thermoacidophilic Sulfolobales. *Environ. Microbiol.* 21 3696–3710. 10.1111/1462-2920.14712 31188531

