# Supplementary material for: Enzymes Catalyzing Crotonyl-CoA Conversion to Acetoacetyl-CoA During the Autotrophic CO2 Fixation in Metallosphaera sedula
Source: Front Microbiol. 2020 Mar 11;11:354. doi: 10.3389/fmicb.2020.00354 (PMC7078158; doi:10.3389/fmicb.2020.00354)
Supplement: Supplementary file 1 [file Data_Sheet_1.PDF]

Supplementary Tables.

Table S1. List of primers used in this study. The restriction enzymes used for the cloning are shown in parentheses, the corresponding restriction sites are underlined.

Primer	Sequence (5'-3')	Annealing temperature	Used for
1423_F	TCAGGATCCGATGCGAATAGCTGTTG (BamHI)	60°C	cloning of *msed_1423*
1423_R	TAGAAGCTTTTAAGTCTTACCGTACGTG (HindIII)		
0389_F	GTTGGATCCGATGAAGGTTTTCGTG (BamHI)	60°C	cloning of *msed_0389*
0389_R	GATAAGCTTTCAATGCACGCCTAG (HindIII)		
0336_F	ATACATATGAAGGTCCTCTACGAGGAAAG (NdeI)	60°C	cloning of *msed_1423*
0336_R	ATAGGATCCTCGTGAAAGGAATCGTTTC (BamHI)		
0384_F	ATACATATGAGAACCGTGATAGTTGAGAAG (NdeI)	60°C	cloning of *msed_0384*
0384_R	ATAGGATCCAGTCTTCCACTCCGGTTTCC (BamHI)		

Table S2. Confirmation of identity of the purified recombinant proteins using gel digestion by trypsin followed by LC-MS/MS.

Protein	Accession	mW (Da)	pI (pH)	PLGS Score	Peptides	Theoretical Peptides	Coverage (%)	Precursor RMS Mass Error (ppm)	Products	Products RMS Mass Error (ppm)	Products RMS RT Error (min)
Msed_0399	ABP94576.1	70823	6.2329	37386.65	644	64	88.9401	2.2036	4730	8.1103	0.01308476
Msed_1423	ABP95581.1	46710	6.6606	48218.89	812	40	99.2806	2.5516	5657	10.1578	0.021885633
Msed_0389	ABP94566.1	40233	6.5361	24202.55	476	41	98.3333	2.8018	2802	8.8158	0.017748512
Msed_0336	ABP94513.1	26463	5.5078	30935.6	24	27	68.6192	2.0569	698	6.4708	0.01928518
Msed_0384	ABP94561.1	30315	8.7554	33238.4	385	29	92.7536	3.0286	2726	9.8104	0.019864192
